# Exploring the Applicability of Pre-Anesthetic Cardiac POCUS in Unexpected Conditions: Could it be Helpful?

**DOI:** 10.24908/pocus.v8i2.16519

**Published:** 2023-11-27

**Authors:** Rodolfo C Sabogal

**Affiliations:** 1 Department of Anesthesiology and Critical Care, Universidad de Cartagena, Universidad de Antioquia Cartagena Colombia

**Keywords:** POCUS, FOCUS, risk assessment, perioperative care, Cardiac POCUS

## Abstract

Formal preoperative echocardiography has traditionally been recommended when there is substantial cardiovascular disease without recent follow up, unexplained dyspnea, a functional class less than 4 METS or a Duke Activity Status Index less than 34. However, it is important to note that certain patients may present with a variety of cardiac abnormalities due to their preexisting condition or multiple treatments, and these individuals warrant consideration. The objective of pre-anesthetic cardiac POCUS is to provide clinical information in a timely manner. Although it does not aim to replace conventional echocardiography, cardiac POCUS can undoubtedly assist anesthesia practitioners in identifying asymptomatic and potentially hazardous conditions, allowing for more accurate risk allocation and individualized patient care.

## Rationale for preoperative cardiac POCUS

Pre-anesthetic evaluation aims to collect data that will enable us to establish rational strategies and enhance interventions to improve care and reduce perioperative risks providing greater safety to the perioperative process. In order to determine if the patient is suitable for anesthesia, typically a series of tests are usually required, including many laboratories and images. Consequently, in place of a one-size-fits-all approach, our objective is to personalize medicine to the greatest extent feasible through the evaluation of each case separately and the provision of a treatment that is tailored to the patient's needs 1. Currently, ultrasonography has experienced greater availability, portability, and cost-effectiveness, enabling health care specialists, including anesthesiologists, to provide meticulous and individualized decisions. In this context, an expert level of ultrasonography understanding and comprehension is necessary in order to incorporate it into daily activities and base actions on the findings 2. 

Cardiac point of care ultrasonography (POCUS) has been designed to provide rapid clinical information 3. Formerly known as focused cardiac ultrasonography (FOCUS), the term cardiac POCUS is now commonly used, yet the terms are frequently interchanged in the literature. Different specialties, including emergency medicine, family medicine, pediatrics, and critical care medicine, among others, have influenced the development and application of this relatively novel technique 4. Cardiac POCUS does not replace formal echocardiography, which usually falls under the scope of cardiologists and cardiac anesthesiologists, but it can be helpful in providing crucial information in the preoperative phase to rapidly evaluate of biventricular dimensions and function, recognize extreme volume states, detect pericardial effusion, and identify morphologic markers of severe valvular disease 5.

Formal preoperative echocardiography has traditionally been recommended when there is substantial cardiovascular disease without recent follow-up, unexplained dyspnea, a functional class less than 4 METS or a Duke Activity Status Index less than 34 6, 7, 8. Nevertheless, cardiac POCUS can aid in the recognition of dangerous, often silent, and unexpected conditions, thereby enabling for improved risk allocation and individualized patient care.

## Evidence supporting preoperative cardiac POCUS

A prospective observational study in which preoperative cardiac POCUS was performed by an anesthesiologist and validated by a cardiologist in 100 patients older than 65 years or with suspected cardiac disease, 54 patients had their anesthetic plan altered; some were referred to a cardiologist, others had their anesthetic or surgical technique altered, and others were referred to highly dependent facilities in the postoperative period 9. In a separate observational study, anesthesiologists performed preoperative cardiac POCUS on 170 patients, obtaining adequate images in 167 (98%) and detecting significant alterations such as mitral valve disease, pulmonary hypertension, and aortic stenosis, pathologies that could alter perioperative management 10, 11. Another prospective observational study involving 99 patients with previous cardiac diagnoses (systolic or diastolic left ventricular failure, vasodilatation, hypovolemia or pericardial effusion) scheduled for non-cardiac emergency surgery, 44% of patients had their treatment modified after preoperative cardiac POCUS 12. An additional prospective cohort study of 100 patients older than 60 years assessed hemodynamics, biventricular function, valvular competence, pericardial effusion, and pulmonary pressures using preoperative cardiac POCUS. They found 26% of patients with LVEF alterations, 18% of patients with valvular lesions, and 40% of patients with diastolic dysfunction. Additionally, the anesthetic behavior of 20% of patients was altered, and 4% of patients were cancelled 13. A separate large database of transthoracic echocardiography (TTE) conducted by the department of anesthesia at the Memorial Sloan Kettering Cancer Center in the United States revealed that cardiac POCUS patterns provided significant responses that guided management in a substantial percentage of patients 14.

Following these observational studies, two randomized controlled trials (RCTs) have recently been conducted. In a pilot RCT led in Australia, anesthesiologists were instructed to perform cardiac POCUS in 100 patients undergoing femoral neck fracture surgery. Cardiac POCUS resulted in a significant decrease in death, acute kidney injury, myocardial infarction, and cerebrovascular accidents 15, 16. In contrast, cardiac POCUS did not appear to reduce postoperative hospitalization days or mortality in the PREOPFOCUS RCT, which included 327 high-risk patients over the age of 65 undergoing abdominal or orthopedic surgery and ASA 3 – 4 17. Unfortunately, this trial had to be terminated early due to COVID-19 restrictions. Perioperative evaluation of cardiac morphology and function is clearly plausible 18. However, conducting real-world studies in the field of pre-operative cardiac POCUS can be challenging due to the heterogeneity of the patient population.

Recent studies indicate that practitioners can comprehend cardiac POCUS after receiving 20 to 30 cases of guided cardiac POCUS education 19. However, it is unknown how many are required for accurate interpretation, though this could be overcome through consistent training. The majority of residency programs in anesthesiology do not include a formal curriculum in the use of perioperative cardiac POCUS, which is typically designated for residents who voluntarily choose to expand their knowledge of cardiac anesthetic or critical care 20. 

## Certain clinical settings in which preoperative cardiac POCUS could be appropriate

### Elderly patients (over 65 years)

The global population is advancing in age and elderly individuals warrant particular attention 21. As a result of aging, geriatric patients experience stiffening of their arterial circulatory system and heart chambers which might result in a variety of illnesses, notably heart failure 22. Valve thickening specifically at mitral or aortic positions (i.e., degenerative, rheumatic, calcific, others) 23, is an additional significant structural change that can occur over time and occasionally lead to stenosis or insufficiencies with potential hemodynamic effects. Arrhythmias are common, especially atrial fibrillation (AF), which worsens with age and may affect 1 in every 5 people by the age of 80, significantly increasing the risk of stroke 24.

Using cardiac POCUS, we can visually determine if the chambers of the heart are enlarged, which indicates the presence of a chronic cardiac condition. Prior to administering anesthesia, a rough estimate of the left ventricle ejection fraction (LVEF) is essential, either qualitatively by eyeballing^25^ or quantitatively using Simpson´s biplane method 26.

In addition, certain findings can aid in determining of an increased thromboembolic risk, predominantly in patients with AF. Left atrial enlargement is a significant indicator of chronic remodeling that provides clues to underlying cardiac disease and is a strong predictor of future events. Using 2D imaging during cardiac POCUS, we will be able to roughly estimate the increase in atrial size, as well as the possible deviation of the interatrial septum and the enlargement of the left atrial appendage 27. There is evidence that a volume increase exceeding 34 ml/m^2^ is associated with an increased risk of ischemic stroke and mortality 28.

Moreover, spontaneous echo contrast resembling the appearance of smoke within the LA indicates blood stasis and stagnation and appears to be a precursor of intracavitary thrombi 29. Reduced velocity (less than 20cm/s) and the presence of a thrombus in the LA appendage are extremely high-risk characteristics; however, transesophageal echocardiography (TEE) is required for detecting them 30. 

Additionally, the assessment of mitral and aortic valves can be performed in order to determine the presence of notable stenosis or regurgitation. However, in these instances, spectral doppler and advanced training are required (Table 1). 

**Table 1:** Preoperative cardiac POCUS for elderly patients.

**Cardiac POCUS utility**	**Critical findings**
· Left ventricular function	· Diastolic and/or systolic dysfunction
· Valvular disease	· Stenosis/regurgitation · Spectral doppler and advance training often required
· Left atrial area and volume	· Severe dilation can be seen in diastolic dysfunction, mitral valve disease

### Patients with cancer

According to the World Health Organization, cancer is the second leading cause of death worldwide, only behind cardiovascular disease 31. This subgroup of patients frequently necessitates preoperative consultation 32.

Patients with cancer may develop a variety of cardiovascular complications, including effects of mediastinal metastatic disease 11, 33, 34, less frequently encountered are primary intracardiac tumors such as sarcomas or myxomas.

Moreover, while new cancer medicines have made remarkable improvements in terms of quality of life and mortality, short- and long-term direct cardiotoxicity is strongly linked to cancer treatment 35, including anthracyclines (i.e. doxorubicin), alkylating agents (i.e. cyclophosphamide), monoclonal antibodies (i.e. trastuzumab, imatinib, bortezomib), among others routinely used in a wide range of hematological and solids tumors. Cancer related coagulation disorders increase the risk of deep vein thrombosis (DVT) and pulmonary embolism (PE) 36, which remain an additional important factor to take into account prior to surgery. 

Cardiac POCUS can play a pivotal role in the necessary preoperative assessment 37. It is crucial to promptly exclude the presence of significant left ventricular (LV) dysfunction (e.g., cardiotoxicity due to chemotherapy). In addition, it is extremely important to look for tumor extension and pericardial effusion, which can go undetected in mild cases and lead to life-threatening tamponade in others.

Although cardiac POCUS does not replace computed tomography in the diagnosis of PE, severe cases can present with acute dilatation and dysfunction of the right ventricle (RV) (low TAPSE and fractional area change [FAC]), and McConnell's sign^38^ (Table 2).

**Table 2:** Preoperative cardiac POCUS for patients with cancer.

**Cardiac POCUS utility**	**Critical findings**
· Left ventricular function	· Diastolic dysfunction and systolic dysfunction due to cardiotoxicity
· Pericardial evaluation	· Pericardial effusion · Tamponade physiology
· Signs of pulmonary embolism	· Right ventricular dilation · Right ventricular disfunction · Ventricular interdependence · Thrombus in transit · McConnell´s sign

### Patients in the intensive care unit 

Critically ill patients are faced with life-threatening multisystem processes and routinely necessitate central vascular access and multiorgan support (e.g., mechanical ventilation and dialysis) 39, which may result in some adverse effects for which cardiac POCUS may be beneficial. Due to the performance of various surgical procedures during their stay in the intensive care unit, this subgroup of patients often requires anesthesiology consultation as part of their multidisciplinary management, and cardiac POCUS can aid cardiovascular evaluation.

Mechanical ventilation support may cause a decrease in RV preload by decreasing right atrial transmural pressure and cause an increase in RV afterload by increasing pulmonary vasculature resistance (e.g., high PEEP), resulting in RV dysfunction in severe cases 40, 41, 42. Cardiac POCUS can also be used to identify increased ventricular interdependence 43.

To evaluate the right atrial (RA) pressure of the patient at the bedside, the diameter of the inferior vena cava (IVC) in proximity to the cavo-atrial junction (CAJ) can be measured 44. Simplified, if the IVC measures less than 10 millimeters with collapse on inspiration, the patient is has low RA pressure (often due to hypovolemia). Conversely, if the IVC exceeds 20 millimeters and does not have respiratory variation, RA pressure is elevated, which can be due to hypervolemia; therefore, perioperative fluid restriction or diuretic use may be required. It is also possible to calculate the velocity time integral (VTI) of the left outflow tract and estimate the cardiac output in order to better comprehend the hemodynamic profile at a fixed point 45, 46. 

Takotsubo syndrome, common but not exclusive to critically ill patients, is a reversible heart failure characterized by apical segment akinesia and basal segment hyperkinesia, resembling the Japanese octopus trap from which it derives its name; however, other atypical patterns can occur. Pre-anesthetic cardiac POCUS can be used to assess left ventricular (LV) function and identify the typical apical-midventricular ballooning pattern, as well as the circumferential pattern of Takotsubo cardiomyopathy 47, 48. 

Cardiac manifestations of septic cardiomyopathy affect 10% to 80% of septic patients 49, involving a variety of patterns ranging from hyperdynamic profiles to biventricular dysfunction; consequently, cardiac POCUS is useful in recognizing some of these features. Catheter-related endocarditis is an uncommon but serious complication associated with central venous catheter infections 50, primarily caused by highly prevalent microorganisms in intensive care units such as staphylococcus aureus and candida spp., hence a high level of suspicion must always be maintained. During the preanesthetic phase, cardiac POCUS has the potential to recognize these endocardial changes (Table 3).

**Table 3:** Preoperative cardiac POCUS for critically ill patients, comorbid patients, and users of illicit psychoactive substances.

**Cardiac POCUS utility**	**Critical findings**
· Right ventricle morphology and function ·	· RV dilation and dysfunction · Tricuspid valve regurgitation jet velocity greater than 2.8m/s, flattened septum, RA enlargement · Ventricular interdependence
· Inferior vena cava assessment	· RA pressure estimate (to aid in assessment of volume status)
· Examination of wall motion	· Global or regional LV dysfunction
· Valve and endocardium morphology	· Valve thickening, masses, thrombi (critically ill, intravenous drug users, comorbid)
· Left atrial dimension	· Diastolic dysfunction parameter
RV: Right ventricle; RA: Right atrial; TAPSE: tricuspid annular plane systolic excursion; VTI: left ventricle outflow tract velocity time integral; LVEF: left ventricle ejection fraction.	

### Patients with comorbidities 

A wide range of diseases, including autoimmune conditions, sickle cell disease, chronic obstructive pulmonary disease, liver cirrhosis, chronic kidney disease, malnutrition, and HIV, among others, may result in latent cardiac conditions. These include pulmonary hypertension with RV compromise (i.e. tricuspid valve regurgitation jet velocity greater 2.8m/s), right atrial and ventricle enlargement, flattened septum caused by volume or pressure overload, cardiomyopathies such as diastolic and systolic biventricular heart failure, pericardial effusions, and other life-threatening complications, many of which are detectable by cardiac POCUS 51, 52, 53.

Coronary artery disease may be suspected if there is the presence of regional wall motion abnormalities, predominantly hypokinesia or akinesia of segments corresponding to a specific vascular territory 54. Furthermore, cardiac POCUS enables a quick estimation of LV systolic function and identification of secondary complications such as free wall or septal rupture, LV aneurysms, intracavitary thrombi, and chronic myocardial remodeling.

### Users of illicit psychoactive substances

Illicit and prohibited substances are widespread in modern society, affecting individuals of all ages, genders, and socioeconomic backgrounds 55. A considerable number of these substances have the potential to directly impair cardiac function. Cocaine is a sympathomimetic substance that can cause long-term myocardial damage due to vasoconstriction, coronary spasm, and platelet aggregation, among other factors. Chronically increased left ventricle mass can result in diastolic heart failure and a significant reduction in LVEF 56. Injection drug-related infective endocarditis can affect individuals who inject drugs 57. It is critical to consider this condition during the pre-anesthetic period. 

## Conclusion

Cardiac POCUS is a rapidly available tool that may assist us in detecting potentially silent and dangerous preoperative conditions, allowing anesthesiologists to provide timely perioperative management. Cardiac POCUS is not intended to replace a formal echocardiogram, traditionally considered the domain of specialists such as cardiologists and cardiac anesthesiologists**. **However, there are situations in which perioperative management requires the assessment of patients for specific conditions that may have immediate cardiac implications.

## Disclosures

Support was provided solely from personal sources. This research did not receive any specific grant from funding agencies in the public, commercial, or not-for-profit sectors.

## References

[R214523329535899] Pelter M N, Druz R S (2022). Precision medicine: Hype or hope?. Trends Cardiovasc Med.

[R214523329535858] Royse C F, Canty D J, Faris J, Haji D L, Veltman M, Royse A (2012). Core Review: Physician-Performed Ultrasound The Time Has Come for Routine Use in Acute Care Medicine. Anesth Analg.

[R214523329535875] Wong A, Chew M, Hernandez G (2023). Using ultrasound in ICU. Intensive Care Med.

[R214523329535893] Johri A M, Glass C, Hill B (2023). The Evolution of Cardiovascular Ultrasound: A Review of Cardiac Point-of-Care Ultrasound (POCUS) Across Specialties. Am J Med.

[R214523329535878] Lenk T, Whittle J, Miller T E, Williams D G A, Bronshteyn Y S (2019). Focused cardiac ultrasound in preoperative assessment: the perioperative provider’s new stethoscope?. Perioper Med.

[R214523329535862] Halvorsen S, Mehilli J, Cassese S (2022). 2022 ESC Guidelines on cardiovascular assessment and management of patients undergoing non-cardiac surgery. Eur Heart J.

[R214523329535879] Smilowitz N R, Berger J S (2020). Perioperative Cardiovascular Risk Assessment and Management for Noncardiac Surgery: A Review. JAMA.

[R214523329535889] Steeds R P, Garbi M, Cardim N (2017). EACVI appropriateness criteria for the use of transthoracic echocardiography in adults: a report of literature and current practice review. Eur Heart J - Cardiovasc Imaging.

[R214523329535868] Canty D J, Royse C F, Kilpatrick D, Bowman L, Royse A G (2012). The impact of focused transthoracic echocardiography in the pre-operative clinic: Transthoracic echocardiography in the pre-operative clinic. Anaesthesia.

[R214523329535888] Cowie B (2011). Three years’ experience of focused cardiovascular ultrasound in the peri-operative period: Focused peri-operative cardiovascular ultrasound. Anaesthesia.

[R214523329535870] D’Cruz I A, Feghali N, Gross C M (1994). Echocardiographic Manifestations of Mediastinal Masses Compressing or Encroaching on the Heart. Echocardiography.

[R214523329535861] Canty D J, Royse C F, Kilpatrick D, Williams D L, Royse A G (2012). The impact of pre-operative focused transthoracic echocardiography in emergency non-cardiac surgery patients with known or risk of cardiac disease: Pre-operative echocardiography in emergency surgery. Anaesthesia.

[R214523329535912] Abdel-Latif A F, Mohamed H S, Ahmed M S, Elzohry A A M (2020). The Role of Pre-Operative Focused Transthoracic Echocardiography in Anesthetic Management of Old Age Patients with Potential Risk of Post-Operative Cardiac Complications. J Anesth Clin Res.

[R214523329535865] Sheth A, Dabo-Trubelja A (2021). Perioperative focused cardiac ultrasound: a brief report. J Anesth Crit Care Open Access.

[R214523329535898] Canty D J, Heiberg J, Yang Y (2019). One-year results of the pilot multicentre randomised trial of preoperative focused cardiac ultrasound in hip fracture surgery. Anaesth Intensive Care.

[R214523329535873] Canty D J, Heiberg J, Yang Y (2018). Pilot multi-centre randomised trial of the impact of pre-operative focused cardiac ultrasound on mortality and morbidity in patients having surgery for femoral neck fractures (ECHONOF-2 pilot). Anaesthesia.

[R214523329535881] Pallesen J, Bhavsar R, Fjølner J (2022). The effects of preoperative focused cardiac ultrasound in high-risk patients: A randomised controlled trial ( PREOPFOCUS ). Acta Anaesthesiol Scand.

[R214523329535891] Barber R L, Fletcher S N (2014). A review of echocardiography in anaesthetic and peri-operative practice. Part 1: impact and utility. Anaesthesia.

[R214523329535880] Millington S J, Hewak M, Arntfield R T (2017). Outcomes from extensive training in critical care echocardiography: Identifying the optimal number of practice studies required to achieve competency. J Crit Care.

[R214523329535863] Mahmood F, Matyal R, Skubas N (2016). Perioperative Ultrasound Training in Anesthesiology: A Call to Action. Anesth Analg.

[R214523329535907] Calcinotto A, Kohli J, Zagato E, Pellegrini L, Demaria M, Alimonti A (2019). Cellular Senescence: Aging, Cancer, and Injury. Physiol Rev.

[R214523329535901] Brunker L B, Boncyk C S, Rengel K F, Hughes C G (2023). Elderly Patients and Management in Intensive Care Units (ICU): Clinical Challenges. Clin Interv Aging.

[R214523329535909] Zern E K, Frank R C, Yucel E (2023). Valvular Heart Disease in the Cardiac Intensive Care Unit. Crit Care Clin.

[R214523329535887] Chyou J Y, Barkoudah E, Dukes J W Atrial Fibrillation Occurring During Acute Hospitalization: A Scientific Statement From the American Heart Association. Circulation.

[R214523329535895] Bergenzaun L, Gudmundsson P, Öhlin H (2011). Assessing left ventricular systolic function in shock: evaluation of echocardiographic parameters in intensive care. Crit Care.

[R214523329535902] Mohsin M, Farooq M U, Akhtar W (2022). Echocardiography in a critical care unit: a contemporary review. Expert Rev Cardiovasc Ther.

[R214523329535884] Inciardi R M, Bonelli A, Biering-Sorensen T (2022). Left atrial disease and left atrial reverse remodelling across different stages of heart failure development and progression: a new target for prevention and treatment. Eur J Heart Fail.

[R214523329535876] Lang R M, Badano L P, Mor-Avi V (2015). Recommendations for Cardiac Chamber Quantification by Echocardiography in Adults: An Update from the American Society of Echocardiography and the European Association of Cardiovascular Imaging. J Am Soc Echocardiogr.

[R214523329535869] Ito T, Suwa M (2019). Left atrial spontaneous echo contrast: relationship with clinical and echocardiographic parameters. Echo Res Pract.

[R214523329535886] Rich M W (1997). Congestive Heart Failure in Older Adults: Epidemiology, Pathophysiology, and Etiology of Congestive Heart Failure in Older Adults. J Am Geriatr Soc.

[R214523329535874] Siegel R L, Miller K D, Wagle N S, Jemal A (2023). Cancer statistics, 2023. CA Cancer J Clin.

[R214523329535856] Gómez-Henao P A, Carreño-Dueñas J A (2016). Cardiovascular pre-anesthesia evaluation in oncological surgery. Colomb J Anesthesiol.

[R214523329535900] Rodríguez C, Fortich F, Quintero E D (2014). Invasión cardiaca de carcinoma sarcomatoide pulmonar a través de las venas pulmonares. Rev Colomb Cardiol.

[R214523329535910] Seca L, Barra S, Matos H (2013). Invasión cardiaca de tumor de esófago. Rev Esp Cardiol.

[R214523329535885] Herrmann J (2020). Adverse cardiac effects of cancer therapies: cardiotoxicity and arrhythmia. Nat Rev Cardiol.

[R214523329535904] Khorana A A, Mackman N, Falanga A (2022). Cancer-associated venous thromboembolism. Nat Rev Dis Primer.

[R214523329535859] Bhasin-Chhabra B, Koratala A (2023). Point of care ultrasonography in onco-nephrology: A stride toward better physical examination. World J Nephrol.

[R214523329535860] Ávila-Reyes D, Acevedo-Cardona A O, Gómez-González J F, Echeverry-Piedrahita D R, Aguirre-Flórez M, Giraldo-Diaconeasa A (2021). Point-of-care ultrasound in cardiorespiratory arrest (POCUS-CA): narrative review article. Ultrasound J.

[R214523329535857] Robertson L C, Al-Haddad M (2013). Recognizing the critically ill patient. Anaesth Intensive Care Med.

[R214523329535896] Zhao Y, Zhang H, Zhang D (2020). Effect of positive end-expiratory pressure on right heart function in mechanically ventilated patients: An ultrasonography based study. KUWAIT Med J.

[R214523329535905] Nieto O R Pérez, López E I Zamarrón, Gutiérrez M A Guerrero, Gutiérrez M A Guerrero (2021). PEEP: dos lados de la misma moneda. Med Crítica.

[R214523329535892] Disselkamp M, Adkins D, Pandey S, Yataco A O Coz (2018). Physiologic Approach to Mechanical Ventilation in Right Ventricular Failure. Ann Am Thorac Soc.

[R214523329535866] Zaidi A, Knight D S, Augustine D X (2020). Echocardiographic Assessment of the Right Heart in Adults: A Practical Guideline from the British Society of Echocardiography. Echo Res Pract.

[R214523329535897] Kearney D, Reisinger N, Lohani S (2022). Integrative Volume Status Assessment. POCUS J.

[R214523329535911] Lescroart M, Pequignot B, Janah D, Levy B (2023). The medical treatment of cardiogenic shock. J Intensive Med.

[R214523329535894] Sattin M, Burhani Z, Jaidka A, Millington S J, Arntfield R T (2022). Stroke Volume Determination by Echocardiography. Chest.

[R214523329535864] Santoro F, Mallardi A, Leopizzi A (2022). Stepwise approach for diagnosis and management of Takotsubo syndrome with cardiac imaging tools. Heart Fail Rev.

[R214523329535883] Citro R, Okura H, Ghadri J R (2020). Multimodality imaging in takotsubo syndrome: a joint consensus document of the European Association of Cardiovascular Imaging (EACVI) and the Japanese Society of Echocardiography (JSE). J Echocardiogr.

[R214523329535872] Carbone F, Liberale L, Preda A, Schindler T H, Montecucco F (2022). Septic Cardiomyopathy: From Pathophysiology to the Clinical Setting. Cells.

[R214523329535871] Chrissoheris M P, Libertin C, Ali R G, Ghantous A, Bekui A, Donohue T (2009). Endocarditis Complicating Central Venous Catheter Bloodstream Infections: A Unique Form of Health Care Associated Endocarditis: Endocarditis complicating CVC-BSI. Clin Cardiol.

[R214523329535890] Kalagara H, Coker B, Gerstein N S (2022). Point-of-Care Ultrasound (POCUS) for the Cardiothoracic Anesthesiologist. J Cardiothorac Vasc Anesth.

[R214523329535908] Rajagopal S, Ruetzler K, Ghadimi K (2023). Evaluation and Management of Pulmonary Hypertension in Noncardiac Surgery: A Scientific Statement From the American Heart Association. Circulation.

[R214523329535882] Chayanupatkul M, Liangpunsakul S (2014). Cirrhotic cardiomyopathy: review of pathophysiology and treatment. Hepatol Int.

[R214523329535867] Esmaeilzadeh M, Parsaee M (2013). The Role of Echocardiography in Coronary Artery Disease and Acute Myocardial Infarction.

[R214523329535903] Prisco L, Sarwal A, Ganau M, Rubulotta F (2021). Toxicology of Psychoactive Substances. Crit Care Clin.

[R214523329535877] Pergolizzi J V, Magnusson P, Lequang Jak, Breve F, Varrassi G (2021). Cocaine and Cardiotoxicity: A Literature Review.. Cureus.

[R214523329535906] Schranz A, Barocas J A (2020). Infective Endocarditis in Persons Who Use Drugs. Infect Dis Clin North Am.

